# High prevalence of sarcopenia and myosteatosis in patients undergoing hemodialysis

**DOI:** 10.3389/fendo.2023.1117438

**Published:** 2023-03-23

**Authors:** Chen Fu, Dong Yan, Ling Wang, Fangfang Duan, Dalong Gu, Ning Yao, Mingke Sun, Di Wang, Xuya Lin, Yanglei Wu, Xiaofei Wang, Xiaoguang Cheng, Dongliang Zhang

**Affiliations:** ^1^ Department of Nephrology, Beijing Jishuitan Hospital, Beijing, China; ^2^ Department of Radiology, Beijing Jishuitan Hospital, Beijing, China; ^3^ Clinical Epidemiology Research Center, Beijing Jishuitan Hospital, Beijing, China; ^4^ MR Collaboration, Siemens Healthineers Ltd., Beijing, China

**Keywords:** hand-grip strength, hemodialysis, muscle cross-sectional area, proton-density fat-fraction, sarcopenia, myosteatosis, quantitative MRI

## Abstract

**Background and purpose:**

Sarcopenia is highly prevalent (28.5–40.3%) in patients undergoing hemodialysis and leads to poor clinical outcomes. However, the association between muscle quality and sarcopenia in patients receiving hemodialysis remains unclear. Therefore, we aimed to explore the association between muscle cross-sectional area (CSA) and proton-density fat-fraction (PDFF) in patients with sarcopenia undergoing hemodialysis.

**Methods:**

Seventy-six patients undergoing hemodialysis for > 3 months were enrolled. Their handgrip strength (HGS), short physical performance battery (SPPB) performance, and appendicular skeletal muscle mass index (ASMI) were measured. Sarcopenia was defined using the Asian Working Group for Sarcopenia 2019 consensus update. All patients underwent quantitative magnetic resonance imaging. CSA and PDFF were measured for the thigh, trunk, and gluteus muscles.

**Results:**

The prevalence of probable, confirmed, and severe sarcopenia in this study was 73.7%, 51.3%, and 22.4%, respectively. Older age (OR: 1.061, *P* < 0.003); lower body mass index (BMI) (OR: 0.837, *P =* 0.008), albumin (OR: 0.765, *P =* 0.004), prealbumin (OR: 0.987, *P =* 0.001), predialysis blood urea nitrogen (BUN) (OR: 0.842, *P <* 0.001), predialysis creatinine (OR: 0.993, *P <* 0.001), phosphorus (OR: 0.396, *P =* 0.047); lower CSA of the thigh (OR: 0.58, *P =* 0.035), third lumbar (L3) trunk (OR: 0.37, *P =* 0.004), gluteus minimus and medius (OR: 0.28, *P =* 0.001), and gluteus maximus (OR: 0.28, *P=* 0.001); and higher PDFF of the thigh (OR: 1.89, *P =* 0.036) and L3 trunk (OR: 1.71, *P =* 0.040) were identified as sarcopenia risk factors. The gluteus minimus and medius CSA was lower in patients with sarcopenia than in those without after adjusting for age and BMI (OR: 0.37, *P =* 0.017). Higher thigh (*P =* 0.031) and L3 trunk (*P =* 0.006) muscle PDFF were significantly associated with lower HGS. Furthermore, higher thigh (*P* = 0.011) and L3 trunk (*P* = 0.010) muscle PDFF were also inversely correlated with lower ASMI.

**Conclusion:**

Our findings demonstrate the high prevalence of sarcopenia and myosteatosis in patients undergoing hemodialysis and might trigger a paradigm shift in intervention strategies for patients receiving hemodialysis.

## 1 Introduction

Sarcopenia is characterized by a gradual decline in physical performance, strength, and skeletal muscle mass (1, 2). It has a prevalence of between 28.5% and 40.3% in patients receiving hemodialysis (3–7) and results in poor clinical outcomes (7–9). The complex pathophysiology of sarcopenia may be exacerbated by metabolic acidosis, oxidative stress, accumulated uremic toxins, inflammation, insulin resistance, malnutrition, protein restriction, decreased appetite, myostatin overexpression, ubiquitination, and physical inactivity (10). Therefore, sarcopenia is a major problem in patients undergoing hemodialysis.

Skeletal muscle mass is the largest component of human free adipose tissue (11). Muscle quality refers to both micro- and macroscopic changes in muscle architecture and composition, and to the amount of function delivered per unit mass of muscle (12). The loss of skeletal muscle mass is one criterion for sarcopenia (1). Moreover, despite the minimal loss in skeletal muscle mass, skeletal muscle function can be drastically reduced with aging (13). This discrepancy may be partially caused by fatty infiltration, which is an aspect of muscle quality. Most studies on sarcopenia in patients undergoing hemodialysis have measured muscle mass using bioelectrical impedance analysis (BIA) (5, 8). However, while BIA is an easy and inexpensive method, it cannot distinguish fat in muscle individually and can therefore not be used to measure muscle fat infiltration (14), usually known as muscle quality. Therefore, whether muscle fat infiltration, namely myosteatosis, exists in patients undergoing hemodialysis remains unclear. Computerized tomography (CT) is an imaging modality that evaluates fat indirectly based on X-ray attenuation (15, 16). However, as CT attenuation values are affected by a variety of factors, including iron, copper, glycogen, fibrosis, and edema, fat quantification is bound to be inaccurate (15). CT scanners manufactured by different vendors demonstrate inherent variations in attenuation values (17). This variability can lead to a platform dependent measurement of fat content, and is thus an important limitation of CT. What is more, participants are exposed to radiation during measurements.

Previous studies (18, 19) suggest that assessing muscle quantity is more important than quantifying muscle mass in the general population. Muscle quantity can be assessed by measuring proton-density fat-fraction (PDFF) using the multi-echo Dixon technique. Recently, assessing fat and water contents has become possible in various body parts through an advanced chemical shift encoding-based water-fat separate magnetic resonance imaging (MRI) approach without invasive quantitative methods (20–22). As a reliable method for quantifying muscle fat infiltration, this method is similar to MR spectroscopy (the “gold” standard) (23, 24); furthermore, the reproducibility of findings produced with this method is high (25).This indicates that PDFFs calculated using the multi-echo Dixon technique accurately reflect fat content.

Typical anatomical locations for skeletal muscle measurements based on computed tomography (CT) are the thigh, hip, and trunk. Additionally, the size and density of the abdominal and thigh muscle bundles are well-established parameters used in cancer studies (26). However, no studies are available on how these muscles contribute to strength and physical performance in patients receiving hemodialysis. Furthermore, no literature has been published on sarcopenia associated with intramuscular adipose tissue in patients undergoing hemodialysis.

Therefore, this study aimed to identify the prevalence of sarcopenia and myosteatosis in patients undergoing hemodialysis and to explore associations among muscle CSA, myosteatosis, and muscle function. We also aimed to identify the clinical and imaging risk factors for sarcopenia in patients undergoing hemodialysis.

## 2 Materials and methods

### 2.1 Study participants

This study was conducted from February 2022 to September 2022. Seventy-six patients undergoing hemodialysis at Beijing Jishuitan Hospital were included. Patients were eligible for inclusion if they were > 18 years of age, had undergone hemodialysis for at least 3 months, three times weekly, on Mondays, Wednesdays, and Fridays, or Tuesdays, Thursdays, and Saturdays, with each session lasting for 3.5–4 h. Exclusion criteria included cognitive or physical disabilities that prevented full participation (e.g., mental retardation, blindness, use of a wheelchair, hand disability, amputated limbs); comorbid medical conditions (e.g., malignant tumors, active inflammatory diseases, pregnancy) or muscular, neuromuscular, or neurologic disorders (e.g., Alzheimer’s or Parkinson’s disease); or antipsychotic medication and corticosteroids use.

This study was conducted in accordance with the principles of the Declaration of Helsinki. The study protocol was reviewed and approved by the Beijing Jishuitan Hospital Ethics Committee (approval number: 202112-11-01). All participants or their legal representatives provided written informed consent. The analyses presented here were based on baseline data taken from a larger, registered trial that can be assessed here: ClinicalTrialsRegistry.gov (NCT05242055).

### 2.2 Clinical and biological parameters

The following clinical variables were recorded: age, sex, cause of renal disease, and dialysis vintage. Anthropometric variables recorded were height, post-dialysis weight, and BMI (dry weight (kg)/height (m)²). The following biological variables were recorded: hemoglobin, serum albumin (bromocresol green method), prealbumin, predialysis blood urea nitrogen (BUN), predialysis creatinine, serum phosphorus, serum bicarbonate, highly sensitive C-reactive protein (hs-CRP; by nephelometry), and dialysis efficacy (Kt/V urea; serum urea was assessed before and after dialysis sessions to calculate urea Kt/V according to the formula of Daugirdas (27)). Laboratory measurements were performed immediately before initiating the Monday or Tuesday hemodialysis session, which was scheduled exactly 68 h after the previous session (i.e., Friday or Saturday). Blood samples were obtained from the central venous catheter, arteriovenous fistula, or graft.

### 2.3 Diagnosis of sarcopenia

#### 2.3.1 Muscle mass

Muscle mass was measured using dual-energy X-ray absorptiometry (DXA) (28). Each patient underwent whole-body DXA scanning (GE Lunar Corp, Madison, WI, USA; software version enCORE-17) at 20–24 h after completing the dialysis session (4). Appendicular skeletal mass (ASM) was calculated as the sum of lean soft tissue from the arms and legs (29). ASM/height^2^ (kg/m^2^) was calculated as the relative ASMI. The Asian Working Group for Sarcopenia (AGWS) 2019 criteria for low muscle mass (low ASMI) in sarcopenia diagnosis are as follows: < 7.0 kg/m^2^ and < 5.4 kg/m^2^ in men and women, respectively (1).

#### 2.3.2 Muscle strength

Muscle strength was assessed based on handgrip strength (HGS) using a Jamar J00105 hydraulic handheld dynamometer. More precisely, HGS was measured in each hand alternately before and after hemodialysis. First, the patient’s arms were placed on armrests while they sat upright in a chair. Next, the elbow of the arm holding the dynamometer was bent at 90° against the patient’s side.

Subsequently, patients were instructed to squeeze the dynamometer’s handle as hard as possible for approximately 3 seconds (30). The highest values of the four trials were recorded. Reduced muscle strength was defined as an HGS measurement of < 28 kg in men and < 18 kg in women (1).

#### 2.3.3 Muscle function

Muscle function was assessed using five-times chair stand time and the SPPB (short physical performance battery), while physical performance was measured the day before the start of the dialysis session. The SPPB assesses lower-body function, including strength, balance, and mobility (31). The SPPB comprises three subtests: five-times repeated chair sit-to-stand [STS time], gait speed [GS], and balance. The balance subtest consisted of three parts, with increasing difficulty levels: unaided feet-together stand, semi-tandem stand, and full-tandem stand. The patients were timed until they moved for 10 seconds. GS was assessed while patients walked 4 meters at their usual pace, with a stationary start. The average time of the two trials was recorded. Patients were asked to fold their arms across their chest and perform five chair stands as quickly as possible to assess their STS time. There were three subtests, each with a score between 0 and 4, and a total score ranging from 0 to 12. Higher SPPB scores indicate better physical function (7). The AWGS 2019 recommends an SPPB total score ≤9 or a five-time STS ≥ 12 seconds as the cut-off for low physical performance (1).

#### 2.3.4 Definition of sarcopenia status

AWGS 19 criteria were adopted for diagnosing sarcopenia (1). First, possible sarcopenia is defined as reduced muscle strength or poor muscle function. Confirmed sarcopenia is defined as reduced low muscle mass and poor muscle function (low STS or SPPB) or low muscle strength. Lastly, severe sarcopenia is characterized by low muscle mass, low strength, and poor muscle function. **Supplementary File S1** shows the details of this classification.

### 2.4 Magnetic resonance data acquisition

On the same day as the DXA examination, the participants underwent a multi-echo 3D spoiled gradient-echo sequence (q-Dixon) for fat fraction quantification using a 3.0-T MRI system (MAGNETOM VIDA, Siemens Healthcare GmbH, Erlangen, Germany). The MRI scanning protocol for participants included axial 2-pt and 6-point (q-Dixon) Dixon scanning of the lumbar spine and thigh. Two-point Dixon scanning was used to obtain a high-resolution anatomical structure, while the q-Dixon scan generated a water image, fat image, T2* map, and PDFF. The total scan time for each patient was 116 seconds. **Supplementary File S2** summarizes the MRI protocols.

### 2.5 Image analyses

**Figure 1:**
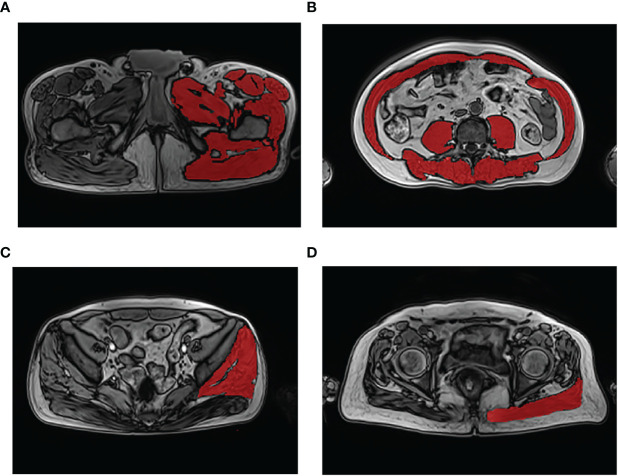
Demonstration of muscle segmentation. Measurement of the left thigh muscle group at the level 3 cm below the lesser trochanter **(A)**; measurement of the trunk muscle at the level of the third lumbar vertebra transverse process **(B)**; measurement of the left gluteus medius and minimus muscles at the third sacral (S3) level **(C)**; measurement of the left gluteus maximus muscle at the level of the greater trochanter of the femur gluteus **(D)**.

The CSA and fat content of the thigh muscles, trunk muscle at the L3 level, gluteus minimus/medius muscle (G. Med/MinM), and gluteus maximus muscle (G. MaxM) were measured. Muscle edges can be visually identified by clear cavities of fat in the muscle. Position criteria for the measurement section are as follows: A) left thigh muscles: 3 cm below the lesser trochanter; B) trunk muscle at the level of the L3 vertebral transverse process; C) left G. Med/MinM muscles at the S3 level; and D) left G.MaxM at the level of the greater trochanter of the femur (**Figure 1**, **Supplementary File S3** show the names of all evaluated muscles).

ITK-SNAP software (Lite version 3.8.0) (32) was used for manual segmentation of the muscle to obtain the muscle area described above. The segmentation was created by a research staff member blinded to participant outcomes. First, DICOM images of the patients were imported into the ITK-SNAP software. Second, muscle labels were measured by manually delineating the region-of-interest (ROI) on the axial T2 images to obtain the CSA value. Once drawn, a radiologist verified the label location and extent to ensure segmentation accuracy. Third, to obtain the PDFF value, the workstation automatically copied the ROI to the fat-fraction map. Finally, the CSA and PDFF of the muscles were automatically calculated using ITK-SNAP software based on measurements taken at the same level in each patient (32).

### 2.6 Statistical analysis

Analyses were performed using the Statistical Package for Social Sciences software (version 21.0; SPSS Inc., Chicago, IL, USA). Statistical significance was set at *P <* 0.05.

Statistical modelling was restricted to confirmed sarcopenia. Continuous variables are presented as either the mean ± standard deviation (SD) or the median and interquartile range. Normality was assessed using the Shapiro–Wilk normality test. Variables were compared between the sarcopenia and non-sarcopenia groups using two-sample *t*-tests or the Mann–Whitney U test. Categorical variables are expressed as absolute (n) and relative frequency (%). Fisher’s exact test was used to analyze categorical variables regarding the primary cause of disease. Other categorical variables were analyzed using chi-square tests. The odds ratios (ORs) and 95% confidence intervals (95% CIs) of non-sarcopenia/sarcopenia were calculated using logistic regression models with and without adjustments for the potential risk factors, with CSA and PDFF levels fitted as continuous variables and results expressed in per-SD increase. Furthermore, the contribution of CSA and PDFF to skeletal muscle mass, strength, and muscle function, with and without adjustments for age, BMI, albumin, predialysis BUN, predialysis creatinine, and phosphorus, was estimated through multivariate linear regression.

## 3 Results

### 3.1 Participant characteristics and prevalence of sarcopenia in patients undergoing hemodialysis

Among the 76 patients on maintenance hemodialysis, 56 (73.7%), 39 (51.3%), and 17 (22.4%) had probable, confirmed, and severe sarcopenia, respectively, according to the AWGS definition. **Table 1** summarizes the baseline characteristics of the 76 patients undergoing hemodialysis. Mean age was 61.8 ± 14.35 years, and 51 (67.1%) participants were male. The causes of end-stage kidney disease included diabetes mellitus (36.8%), chronic glomerulonephritis (22.4%), hypertension nephrosclerosis (25%), and other nephropathies (15.7%). A low ASMI, low HSG, slow five-time STS, and low SPPB scores were observed in 64.5%, 48.7%, 50%, and 60.5% of all patients, respectively.

**Table 1:** Characteristics of the hemodialysis patients in the sarcopenia and the non-sarcopenia group.

Characteristics	Total	Non-sarcopenia	Sarcopenia	*P*
	(n=76)	n=37	n=39	
Age, years	61.8 (14.35)	56.46 (13.85)	66.87 (12.12)	**0.001**
Sex (male), n (%)	51 (67.1%)	28 (75.6%)	23 (58.9%)	0.121
Dialysis vintage, Mo	8.00 (4.00-20.5)	10.00 (7.00-48.50)	6.00 (4.00-9.00)	**0.002**
Primary cause of disease				
Diabetic nephropathy, n (%)	28 (36.8%)	9 (24.3%)	19 (48.7%)	**0.014**
Chronic glomerulonephritis, n (%)	17 (22.4%)	12 (32.4%)	5 (12.8%)	**0.037**
Hypertensive nephrosclerosis n (%)	19 (25%)	9 (24.2%)	10 (25.6%)	0.553
Polycystic kidney disease, n (%)	4 (5.3%)	2 (5.4%)	2 (5.1%)	0.672
Interstitial nephritis, n (%)	3 (3.9%)	2 (5.4%)	1 (2.6%)	0.48
Lupus nephritis, n (%)	2 (2.6%)	0 (0%)	2 (5.1%)	0.26
Obstructive nephropathy, n (%)	3 (3.9%)	3 (8.1%)	0 (0%)	0.111
Anthropometry measures				
Weight, kg	63.75 (13.40)	68.96 (12.32)	58.81 (12.61)	**0.001**
Height, cm	165.54 (9.22)	167.68 (8.31)	163.51 (9.68)	**0.048**
BMI, kg/m^2^	23.15 (3.96)	24.43 (3.45)	21.94 (4.07)	**0.005**
Skeletal muscle measures				
ASM, kg	18.19 (14.03,21.17)	20.31 (17.30,23.19)	15.14 (11.89,21.32)	**0.004**
ASMI	6.01 (1.09)	6.77 (0.85)	5.30 (0.76)	**<0.001**
Male		7.14 (0.51)	5.51 (0.71)	**<0.001**
Female		5.59 (0.57)	4.99 (0.74)	**0.036**
Handgrip strength, kg	23.86 (11.15)	29.81 (10.99)	18.22 (7.99)	**<0.001**
Male		33.75 (8.98)	21.54 (8.04)	**<0.001**
Female		17.56 (6.97)	13.44 (5.07)	0.102
Slow 5- time chair stand test, n (%)	46 (60.5%)	22 (59.5%)	24 (61.5%)	0.853
Low SPPB score, n (%)	38 (50.0%)	8 (21.6%)	30 (76.9%)	**<0.001**
Laboratory data				
Hemoglobin, g/L	106.16 (18.71)	107.92 (16.23)	104.49 (20.87)	0.425
Albumin, g/L	36.18 (3.56)	37.51 (2.17)	34.92 (2.13)	**0.001**
prealbumin, mg/L	268.33 (80.57)	302.76 (59.25)	235.68 (85.078)	**<0.001**
Predialysis BUN (mmol/L)	22.32 (6.27)	25.13 (5.90)	19.66 (5.45)	**<0.001**
Predialysis Creatinine(µmol/L)	811.09 (302.00)	997.41 (256.00)	634.34 (228.17)	**<0.001**
Phosphorus, mmol/L	1.63 (0.53)	1.75 (0.51)	1.51 (0.54)	
Bicarbonate, mmol/L	21.42 (3.02)	21.07 (0.17)	**21.76 (0.77)**	0.321
hs-CRP, mg/L	2.38 (1.28,5.83)	2.03 (1.20-5.85)	2.72 (1.59-5.81)	0.199
Kt/V	1.41 (0.24)	1.37 (0.26)	1.44 (0.23)	0.208
Muscle measurement by MRI				
Thigh muscle CSA (mm^2^)	8559.82 (1656.81)	8985.42 (1650.33)	8156.05 (1579.2)	**0.028**
L3 trunk muscle CSA (mm^2^)	9493.45 (8299.73,10094.9)	9592.4 (9019.55,11920.6)	9409.13 (8082.2,9649.52)	**0.015**
G.Med/MinM CSA (mm^2^)	2979.33 (2584.15,3204.6)	3012.73 (2926.76,3860.15)	2952.08 (2025,2989.15)	**<0.001**
G.MaxM CSA (mm^2^)	2799.33 (2514.94,3153.68)	2862.09 (2761.07,3607.03)	2773.42 (2171.34,2827.91)	**<0.001**
Thigh muscle PDFF (%)	10.56 (8.61,11.94)	9.92 (7.65,10.94)	11.29 (10.28,13.76)	**0.001**
L3 trunk muscle PDFF (%)	13.51 (3.05)	10.13 (3.95)	12.13 (35.95)	**0.033**
G.Med/MinM PDFF (%)	14.39 (14.39,15.56)	14.23 (10.92,15.81)	14.47 (14.02,15.47)	0.257
G.MaxM PDFF (%)	15.47(13.65,16.09)	14.68(11.28,15.92)	15.73(15.09,16.59)	**0.015**

Data are expressed as numbers, percentages, mean ± standard deviation, or median (interquartile range).For the comparisons between groups, t-tests were used for normally distributed and the Mann-Whitney U test for skewed variables. Fisher’s exact test was used to analyze categorical variables regarding the primary cause of disease. Other categorical variables were analyzed using chi-square tests. Values of P < 0.05 are marked in bold.

BMI, body mass index; ASM, appendicular skeletal muscle mass; ASMI, ASM index (ASM/height2); SPPB, short physical performance battery; BUN, blood urea nitrogen; hs-CRP, high-sensitive C-reactive protein; Kt/V, dialysis efficacy; L3 trunk, third lumbar trunk; G.Med/MinM, gluteus medius and minimus muscles; G.MaxM, gluteus maximus muscle; CSA, muscle cross-sectional area; PDFF, proton-density fat-fraction.

### 3.2 Risk factors for sarcopenia in patients undergoing hemodialysis

**Table 1** shows differences in hemodialysis status between the patients with sarcopenia and those without. Patients with sarcopenia were older (*P* < 0.001); had a lower BMI (*P =* 0.005), weight (*P =* 0.001), height (*P =* 0.048), ASM (*P <* 0.001), ASMI (*P <* 0.001), HGS (*P <* 0.001), SPPB score (*P <* 0.001), albumin (*P =* 0.001), prealbumin (*P* = 0.001), predialysis BUN (*P <* 0.001), predialysis creatinine (*P <* 0.001), phosphorus (*P =* 0.042), thigh muscle CSA (*P =* 0.028), L3 trunk muscle CSA (*P =* 0.015), G. Med/MinM CSA (*P <* 0.001), and G. MaxM CSA (*P <* 0.001); they also exhibited a higher dialysis vintage in **Supplementary File S5** (*P =* 0.002), and a higher PDFF of the thigh muscle (*P =* 0.001), L3 trunk muscle (*P =* 0.033) and G. MaxM (*P =* 0.015).

**Supplementary File S4** shows the risk factors for sarcopenia in patients receiving hemodialysis. Older age (OR: 1.061, *P* < 0.003); lower BMI (OR: 0.837, *P =* 0.008), albumin (OR: 0.765, *P =* 0.004), prealbumin (OR: 0.987, *P =* 0.001), BUN (OR 0.842, *P <* 0.001), predialysis creatinine (OR: 0.993, *P <* 0.001), and phosphorus (OR: 0.396, *P =* 0.047); and a higher PDFF of the thigh muscle (OR: 1.89, *P =* 0.036) and the L3 trunk muscle (OR: 1.71, *P =* 0.040) were identified as sarcopenia risk factors.

### 3.3 Associations of the CSA and PDFF with sarcopenia in patients undergoing hemodialysis

**Figure 2:**
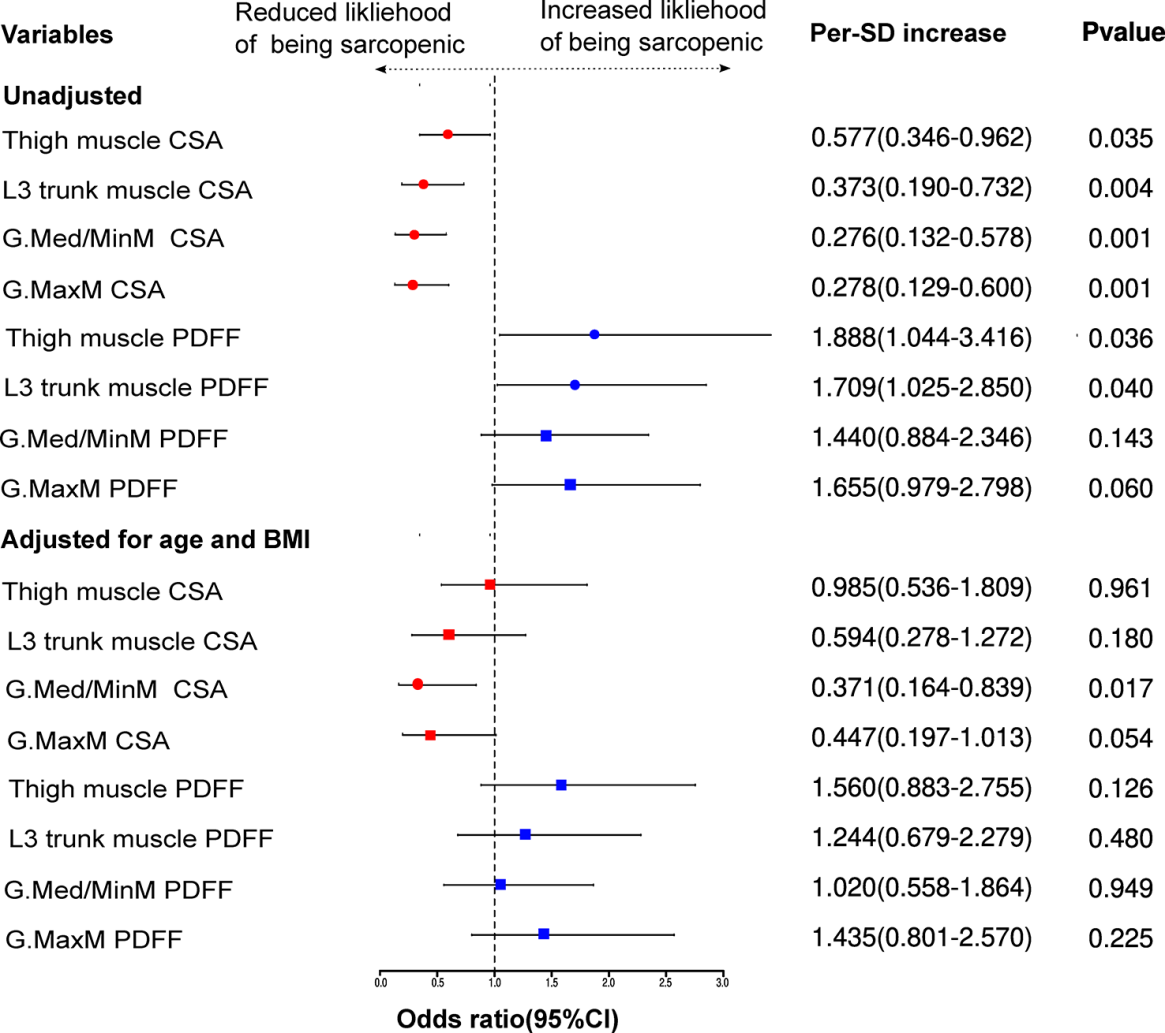
Association of muscle measurements with sarcopenia in hemodialysis patients. P < 0.05 was considered statistically significant. CI, confidence interval; L3 trunk, third lumbar trunk; G.Med/MinM, gluteus medius and minimus muscles; G.MaxM, gluteus maximus muscle; PDFF, proton-density fat-fraction. circle = significant; square = non-significant.

Logistic regression showed that muscle CSA and PDFF were significant predictors of sarcopenia among patients undergoing hemodialysis in this study, as shown in **Supplementary File S5** and **Figure 2**. Except for the PDFF of G.MaxM and G. Med/MinM, the CSA and PDFF of the other muscles correlated with sarcopenia. For a per-SD increase in the CSA of G. Med/MinM, the OR for sarcopenia was 0.371 (95% CI: 0.164–0.839) in a multivariable model adjusted for age and BMI.

### 3.4 Association of the CSA and PDFF with muscle function measures

**Tables 2**–**4** present the simple and multiple linear regression analyses of CSA and PDFF concerning HGS, SPPB score, and ASMI, respectively. In the unadjusted model (model 1), the muscle CSA and PDFF were significantly associated with HGS. Except for the L3 trunk muscle CSA, the CSA and PDFF of the other muscles were significantly associated with the SPPB score. Additionally, the CSA and PDFF of the other muscles were significantly associated with ASMI, excluding the G. Med/MinM PDFF and G. MaxM PDFF. After adjustments for age, BMI, albumin, predialysis BUN, predialysis creatinine, and phosphorus (model 5), a lower HGS was associated with lower thigh muscle CSA (ß = 0.267, *P* = 0.018) and G. Med/MinM CSA (ß = 0.280, *P <* 0.020), and a higher PDFF of the thigh muscle (ß = -0.209, *P =* 0.031) and L3 trunk muscle (ß =-0.287, *P =* 0.006). However, the muscle CSA and PDFF were not associated with the SPPB score after adjustments for age, BMI, albumin, predialysis BUN, predialysis creatinine, and phosphorus (model 5). Contrary to the SPPB results, in model 5, a lower ASMI was associated with a lower CSA of the thigh muscle (ß = 0.364, *P =* 0.001), L3 trunk muscle (ß = 0.428, *P <* 0.001), G. Med/MinM (ß = 0.342, *P =* 0.003), and G. MaxM (ß = 0.315, *P =* 0.004). Moreover, a lower ASMI was also associated with a higher PDFF of the thigh muscle (ß = -0.236, *P =* 0.011) and L3 trunk muscle (ß = -0.259, *P =* 0.010).

**Table 2:** Independency of muscle measurements and handgrip strength.

	HGS (kg)									
Measurement	model 1		model 2		model 3		model 4		model 5	
	β	*P*	β	*P*	β	*P*	β	*P*	β	*P*
Thigh muscle CSA (mm^2^)	0.503	**<0.001**	0.483	**<0.001**	0.433	**<0.001**	0.382	**0.001**	0.267	**0.018**
L3 trunk muscle CSA (mm^2^)	0.518	**<0.001**	0.504	**<0.001**	0.448	**<0.001**	0.402	**0.001**	0.227	0.062
G.Med/MinM CSA (mm^2^)	0.552	**<0.001**	0.543	**<0.001**	0.490	**<0.001**	0.454	**<0.001**	0.280	**0.020**
G.MaxM CSA (mm^2^)	0.383	**0.001**	0.349	**0.005**	0.304	**0.007**	0.221	0.079	0.079	0.500
Thigh muscle PDFF (%)	-0.381	**0.001**	-0.372	**0.001**	-0.317	**0.004**	-0.300	**0.005**	-0.209	**0.031**
L3 trunk muscle PDFF (%)	-0.443	**<0.001**	-0.447	**<0.001**	-0.353	**0.003**	-0.344	**0.003**	-0.287	**0.006**
G.Med/MinM PDFF (%)	-0.325	**0.004**	-0.337	**0.002**	-0.202	0.100	-0.203	0.086	-0.187	0.073
G.MaxM PDFF (%)	-0.351	**0.002**	-0.378	**0.001**	-0.259	**0.024**	-0.281	**0.011**	-0.194	0.053

β is the regression coefficient. Values of P < 0.05 are marked in bold.

L3 trunk, third lumbar trunk; G.Med/MinM, gluteus minimus and medius muscle; G.MaxM, gluteus maximus muscle; CSA, muscle cross-sectional area; PDFF, proton-density fat-fraction.

model 1, unadjusted; model 2, adjusted for BMI; model 3, adjusted for age; model 4, adjusted for age and BMI; model 5, adjusted for age, BMI, albumin, predialysis BUN, predialysis creatinine, and phosphorus.

**Table 3:** Independency of muscle measurements and SPPB score.

	SPPB score									
Measurement	model 1		model 2		model 3		model 4		model 5	
	β	*P*	β	*P*	β	*P*	β	*P*	β	*P*
Thigh muscle CSA (mm^2^)	0.165	0.161	0.150	0.236	0.015	0.888	-0.064	0.593	-0.128	0.279
L3 trunk muscle CSA (mm^2^)	0.194	0.097	0.185	0.146	0.036	0.744	-0.045	0.712	-0.170	0.171
G.Med/MinM CSA (mm^2^)	0.365	**0.001**	0.388	**0.002**	0.242	**0.024**	0.213	0.076	0.087	0.488
G.MaxM CSA (mm^2^)	0.250	**0.032**	0.258	**0.048**	0.116	0.286	0.047	0.706	-0.064	0.595
Thigh muscle PDFF (%)	-0.254	**0.029**	-0.251	**0.032**	-0.154	0.145	-0.144	0.172	-0.074	0.460
L3 trunk muscle PDFF (%)	-0.284	**0.014**	-0.286	**0.014**	-0.078	0.502	-0.072	0.536	-0.068	0.535
G.Med/MinM PDFF (%)	-0.321	**0.005**	-0.331	**0.004**	-0.126	0.280	-0.131	0.257	-0.121	0.258
G.MaxM PDFF (%)	-0.318	**0.006**	-0.331	**0.004**	-0.172	0.117	-0.183	0.093	-0.161	0.116

β is the regression coefficient; The values of P < 0.05 were marked in bold.

L3 trunk, third lumbar trunk; G.Med/MinM,gluteus minimus and medius muscle; G.MaxM, gluteus maximus muscle; CSA, muscle cross-sectional area; PDFF, proton-density fat-fraction.

model 1, Unadjusted. model 2, Adjusted for BMI. model 3, Adjusted for age. model 4, Adjusted for age and BMI. model 5, Adjusted for age, BMI, albumin, predialysis BUN, predialysis creatinine, and phosphorus.

**Table 4:** Independency of muscle measurements and ASMI.

	ASMI (kg/m^2^)									
Measurement	model 1		model 2		model 3		model 4		model 5	
	β	*P*	β	*P*	β	*P*	β	*P*	β	*P*
Thigh muscle CSA (mm^2^)	0.550	**<0.001**	0.414	**<0.001**	0.573	**<0.001**	0.407	**<0.001**	0.364	**0.001**
L3 trunk muscle CSA (mm^2^)	0.635	**<0.001**	0.509	**<0.001**	0.674	**<0.001**	0.525	**<0.001**	0.428	**<0.001**
G.Med/MinM CSA (mm^2^)	0.602	**<0.001**	0.471	**<0.001**	0.622	**<0.001**	0.468	**<0.001**	0.342	**0.003**
G.MaxM CSA (mm^2^)	0.569	**<0.001**	0.420	**<0.001**	0.587	**<0.001**	0.411	**<0.001**	0.315	**0.004**
Thigh muscle PDFF (%)	-0.363	**0.001**	-0.341	**<0.001**	-0.356	**0.002**	-0.321	**0.001**	-0236	**0.011**
L3 trunk muscle PDFF (%)	-0.305	**0.007**	-0.313	**0.001**	-0.323	**0.012**	-0.303	**0.005**	-0.259	**0.010**
G.Med/MinM PDFF (%)	-0.221	0.056	-0.246	**0.012**	-0.216	0.098	-0.218	**0.048**	-0.193	0.055
G.MaxM PDFF (%)	-0.193	0.095	-0.248	**0.012**	-0.176	0.154	-0.218	**0.036**	-0.156	0.109

β is the regression coefficient. Values of P < 0.05 are marked in bold.

L3 trunk, third lumbar trunk; G.Med/MinM, gluteus minimus and medius muscle; G.MaxM, gluteus maximus muscle; CSA, muscle cross-sectional area; PDFF, proton-density fat-fraction.

model 1, unadjusted. model 2, adjusted for BMI. model 3, adjusted for age. model 4, adjusted for age and BMI. model 5, adjusted for age, BMI, albumin, predialysis BUN, predialysis creatinine, and phosphorus.

## 4 Discussion

In this study, we identified probable, confirmed, and severe sarcopenia in 73.7%, 51.3%, and 22.4% of patients undergoing hemodialysis, respectively. Older age; lower BMI, albumin, prealbumin, predialysis BUN, predialysis creatinine, and phosphorus levels; lower CSA of the thigh muscle, L3 trunk muscle, G. Med/MinM, and G. MaxM; and a higher PDFF of the thigh and L3 trunk muscle were identified as sarcopenia risk factors. G. Med/MinM CSA was higher in those without sarcopenia after adjusting for age and BMI. The lower thigh and G. Min/Med muscle CSA, as well as the higher thigh and L3 trunk muscle PDFF, were associated with lower HGS after adjustments for known risk factors. Moreover, a higher thigh and L3 trunk muscle PDFF inversely correlated with a lower ASMI.

In a recent meta-analysis including studies with 692 056 participants, the prevalence of sarcopenia in the general population was approximately 10.0%–27.0% (33). Because of the co-existence of factors such as uremic toxins, insulin resistance, or oxidative stress in patients with renal failure (34), they are more likely to develop sarcopenia. Shu et al. (7) recently published a meta-analysis showing that the sarcopenia prevalence was 28.5% (95% CI: 22.9–34.1%) and varied from 25.9% (I^2^ = 94.9%, 95% CI: 20.4–31.3%; combined criteria) to 34.6% (I^2^ = 98.1%, 95% CI: 20.9–48.2%; low muscle mass alone) in patients receiving hemodialysis almost two times the prevalence observed in patients without chronic kidney disease (CKD). Interestingly, we found similar results. The prevalence of confirmed sarcopenia in our study was 51.3%, based on the AWGS (2019) definition, and the prevalence of sarcopenia in our patients receiving hemodialysis was higher than that in patients undergoing hemodialysis in previous studies (7). This difference may be due to the following reasons: First, the populations selected differed (hospitalized and outpatients). Second, different instruments were used to assess muscle mass (DXA, bioelectrical impedance analysis, magnetic resonance imaging, and body composition monitors). DXA is the “gold” standard, and other detection methods may overestimate muscle mass due to overhydration in patients undergoing hemodialysis (35). Third, the difference may be due to the large variability in diagnostic criteria, such as those propsed by the European Working Group on Sarcopenia in Older People, the AWGS, the Foundation for the National Institutes of Health Sarcopenia Project, and the International Working Group on Sarcopenia.

The pathogenesis of sarcopenia remains unclear, and only a few reports have discussed the pathogenesis of sarcopenia in patients receiving hemodialysis. To identify the risk factors of sarcopenia in such patients, we categorized our sample into two groups, one with sarcopenia and one without. Our analyses show that older age was a risk factor for sarcopenia in our patients undergoing hemodialysis, which is consistent with previous results in the general population as well as in patients receiving hemodialysis (4, 6), and may be related to alpha motor neuron loss caused by aging (36). Furthermore, we found that a higher BMI and predialysis BUN and higher serum albumin, prealbumin, and phosphate levels were sarcopenia-protective factors in our patients receiving hemodialysis. These results are in agreement with previous findings reported in the literature (4, 5, 9). The reduction in the abovementioned indicators is reflective of poor oral intake, malnutrition, and poor nutritional status (37), which may result in reduced protein synthesis and muscle weakness (38). Therefore, it is appropriate to implement precise nutritional measures for patients undergoing hemodialysis.

A low predialysis creatinine level was found as a risk factor for sarcopenia in patients undergoing hemodialysis in our study. This marker is influenced by muscle mass (6, 9). We identified that the primary cause of the disease was diabetic nephropathy, which was significantly associated with sarcopenia in our patients receiving hemodialysis, in agreement with the literature (38, 39).

Some studies have found no statistically significant association between dialysis vintage and sarcopenia (5, 6, 9). However, a lower dialysis vintage was associated with sarcopenia in our study. We considered the following reason as the cause for this finding: most of our patients had a low dialysis vintage (in 71% of cases, the dialysis vintage was < 12 months); therefore, the dialysis-related indicators may not have had time to develop. Previous studies have reported that sarcopenia in patients receiving hemodialysis is mainly associated with hs-CRP (39), hemoglobin (6), and Kt/V (38). No correlations were found between these indicators and sarcopenia, which may be due to the limited sample as well as to the fact that patients with good control of the above indicators were under dialysis care and receiving a pharmacological intervention.

An individual’s muscle quality can be measured by both micro- and macro-scale changes in muscle architecture and composition, as well as by muscle function per unit of muscle mass (12).. Various imaging techniques, including CT and MRI, have been used to assess muscle quality in study settings, including the measurement of fat infiltration and muscle attenuation (40, 41). There is a strong association between fatty infiltration in the muscles and reduced muscle function (18). Several pathways contribute to the accumulation of fatty acids in muscles, and the accumulation of lipids within myofibers, also known as intramuscular fat, is one direct route. Adipocytes, which accumulate within the skeletal muscle as intermuscular fat, represent another pathway (42). Here, we used MR Dixon technology to quantify the amount of muscle adipose tissue, including intramuscular and intermuscular adipose tissue.

In addition to assessing the traditional risk factors for sarcopenia in patients undergoing hemodialysis, we evaluated new muscle measurements, such as CSA and PDFF. To our knowledge, no previous study has compared CSA and PDFF findings between patients with and without sarcopenia using quantitative MRI scans in those receiving hemodialysis. Using quantitative MRI, our study showed that muscle CSA reduction (thigh muscle, L3 trunk muscle, G.MaxM, and G. Med/MinM) and PDFF increase (thigh muscle, L3 trunk muscle, and G.MaxM) were influencing factors of sarcopenia in patients undergoing hemodialysis. Further analysis using binary logistic regression showed that the muscle CSA reduction (thigh muscle, L3 trunk muscle, G.MaxM, and G.Med/MinM) and PDFF increase (thigh muscle, L3 trunk muscle, and G.MaxM) were risk factors for developing sarcopenia. Our analyses demonstrate that the CSA reduction of G.MaxM and G.Med/MinM independently predicted sarcopenia, even after adjusting for age and BMI. Studies conducted as part of the Health ABC project have also shown age-related decreases in thigh muscle density or increased fatty infiltration in the thigh muscle (43), which may explain why some indicators were not statistically significant after age adjustments. After adjustments for age, BMI, albumin, predialysis BUN, predialysis creatinine, and phosphorus, muscle CSA and PDFF were not significantly associated with sarcopenia, which may partially be explained by low power due to the limited sample size, or by direct effects of chronic kidney disease on muscle function.

The assessment of muscle quality is expected to guide treatment decisions. Therefore, we assessed muscle measurements (CSA and PDFF) and muscle characteristics (muscle mass, strength, and function). We found that low muscle CSA and high PDFF were risk factors for lower muscle strength. After full adjustments for age, BMI, and other laboratory risk factors, CSA reduction (thigh muscle, L3 trunk muscle, and G.MaxM) and PDFF increase (thigh muscle, L3 trunk muscle, and G.Med/MinM) were also identified as risk factors for lower muscle strength. Some previous studies have reached similar conclusions, and muscle strength can be indirectly measured using muscle volume (26, 44, 45). However, Wang et al. (18) found no associations between HGS and midthigh muscle variables (neither muscle area nor density).

Our study suggests that CSA reduction (G. Med/MinM and G.MaxM) and PDFF increase (thigh muscle, L3 trunk muscle, G. Med/MinM, and G.MaxM) are also risk factors for lower muscle function. Particularly, after adjusting for BMI, CSA reduction (G. Med/MinM and G.MaxM) and PDFF increase (thigh muscle, L3 trunk muscle, G. Med/MinM, and G.MaxM) were identified as risk factors for lower muscle function. Our results confirm that muscle CSA and PDFF are important parameters for detecting lower muscle function, which is in line with other studies (16, 46). The fat fraction in the thigh muscle was associated with performance in a timed up-and-go test after adjusting for muscle area in controlled acromegaly (47). Moreover, older adults with low trunk muscle density are more likely to have poor balance and exhibit faster declines in functional capacity (16). Anderson et al. (16) found no association between L2 trunk muscle volume and the SPPB score, as well as between L2 trunk muscle density and the SPPB score, which is inconsistent with our results. Our findings show that only the G. Med/MinM CSA predicted lower muscle function after adjusting for age and BMI. Additionally, we identified low G. Med/MinM CSA as the most sensitive indicator of lower muscle function. Other studies reported similar results: Andrew et al., for example, found that normal gait is largely influenced by the G. Med/Min muscles of the hip (48). The G. Med/MinM, which is known as the “rotator cuff of the hip,” inserts into the greater trochanter of the femur. It maintains balance by acting as an abductor and rotator of the hip during normal walking (49). Similarly, a recent study found that stair climbing, sitting up and standing down, and walking are associated with smaller gluteus muscles (50). Therefore, the G. Med/MinM may be a key muscle group to focus on in future studies.

This study has some limitations. First, since this was a single-center study conducted in China, the results may not be generalizable to other patient populations and countries. Second, this study had a cross-sectional design, which prevented us from analyzing causal relationships between sarcopenia and muscle measurements (CSA and PDFF) in hemodialysis patients. Third, a limited patient sample was enrolled in this study, and some indicators were not found to influence sarcopenia, which may have been due to the resulting low power. Fourth, as image registration was not applied in this study, the absolute values of the cross-sectional area could have been affected by slight changes in image orientation. Fifth, some potentially relevant information was not collected, such as details on peripheral vascular disease, physical activity, nutritional status, and some muscle-affecting medicines (e.g., those belonging to the statin family). Therefore, a multicenter study with a larger sample size would be beneficial in evaluating this issue in the future.

In conclusion, our findings demonstrate the value of fat content assessments within skeletal muscles in patients with sarcopenia undergoing hemodialysis and might trigger a paradigm shift in intervention strategies for sarcopenia. In the future, assessments of muscle fat infiltration and muscle CSA may help guide treatment choices (i.e., precise nutritional exercise interventions) and monitor treatment responses.

## Data availability statement

The raw data supporting the conclusions of this article will be made available by the authors, without undue reservation.

## Ethics statement

The studies involving human participants were reviewed and approved by the Beijing Jishuitan Hospital Ethics Committee (ethics committee approval number: 202112-11-01). The patients/participants provided their written informed consent to participate in this study.

## Author contributions

XC, DZ, CF, and LW designed the study. CF and DY prepared the first draft of the paper. NY, LW, MS, DW, XL, and XW contributed to the data collection, such as information collection, scanning, and data input. DY and YW edited the draft. FD and CF were responsible for the statistical analysis of the data. CF, DZ, and LW supervised the study and paper organization. All authors contributed to the article and approved the submitted version.
