# Supplementary material for: High prevalence of sarcopenia and myosteatosis in patients undergoing hemodialysis
Source: Front Endocrinol (Lausanne). 2023 Mar 23;14:1117438. doi: 10.3389/fendo.2023.1117438 (PMC10076821; doi:10.3389/fendo.2023.1117438)
Supplement: Supplementary file 1 [file DataSheet_1.docx]

Supplementary File S1

Asian Working Group for Sarcopenia (AWGS) 2019 conceptual stages of sarcopenia.

| **Stage** | **Muscle strength** | |  | **Muscle performance** | | |  | **Muscle mass** | |  |  |
| --- | --- | --- | --- | --- | --- | --- | --- | --- | --- | --- | --- |
| **Possible sarcopenia** | Low muscle strength | | **or** | Low physical performance | | |  |  |  |  |  |
|  | Handgrip strength | |  | 5-time chair stand test (≥12 s) | | |  |  |  |  |  |
|  | (M: <28 kg, F: <18 kg) | |  |  |  |  |  |  |  |  |  |
| **Confirmed sarcopenia** |  |  |  |  |  |  |  |  |  |  |  |
| **Sarcopenia** | Low muscle strength | | **add** | Low ASMI |  |  | **or** | Low physical performance | | |  |
|  | Handgrip strength | |  | Dual-energy X-ray absorptiometry | | | | 6-meter walk: <1.0 m/s | | |  |
|  | (M: <28 kg, F: <18 kg) | | | (M: <7.0 kg/m2, F: <5.4 kg/m2) | | |  | or 5-time chair stand test: ≥12 s | | | |
|  |  |  |  |  |  |  |  | or short physical performance battery: ≤9 | | | |
| **Severe sarcopenia** | Low muscle strength | | **add** | Low ASMI |  |  | **add** | Low physical performance | | |  |

ASMI: appendicular skeletal muscle mass index

| Supplementary File S2. MRI scan parameters. |  |  |  |  |  |  |  |
| --- | --- | --- | --- | --- | --- | --- | --- |
| \| **Images** \| **Coil** \| **TR (ms)** \| **TE (ms)** \| **Voxel size (mm^3^)** \| **Matrix Size(m^2^)** \| **Average NEX** \| **FOV (mm)** \| **ACQ Time(s)** \| **ST (mm)** \| **Bandwidth** \| \| --- \| --- \| --- \| --- \| --- \| --- \| --- \| --- \| --- \| --- \| --- \| \| 2-pt Dixon \| Flex Large 18 Body 18 Spine 32 \| 3.88 \| 1.23/2.46 \| 1.4x1.4x3.0 \| 1.4x1.4 \| 1 \| 450x84.4% \| 12 \| 3 \| 1040 Hz/Px \| \| 6-pt Dixon \| 8.82 \| 1.05/2.46/3.69/4.92/6.15/7.38 \| 1.4x1.4x3.0 \| 1.4x1.4 \| 1 \| 450x87.5% \| 17 \| 3 \| 1080 Hz/Px \|   TR, time of repetition; TE, echo time; NEX, number of excitations; FOV, field of view; ACQ, acquisition; ST, slice thickness | | | | | | | |

Supplementary File S3.

Names of all evaluated muscles.


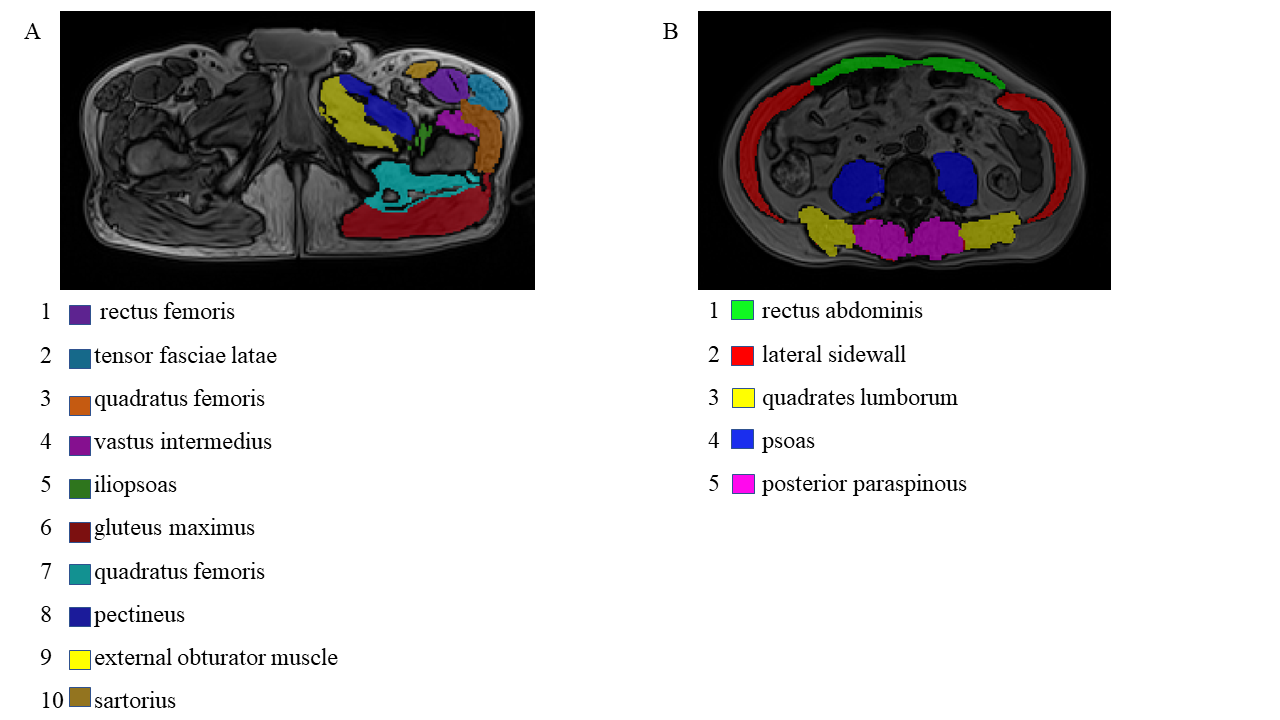


| Supplementary File S4. Associations of risk factors with sarcopenia in patients undergoing hemodialysis. | | | |
| --- | --- | --- | --- |
| **Characteristics** | **OR** | **95%CI of OR** | ***P* value** |
| Age, years | 1.061 | (1.020,1.103) | 0.003 |
| Sex (male), n (%) | 2.164 | (0.808,5.798) | 0.125 |
| Dialysis vintage, Mo | 0.97 | (0.945,0.996) | 0.023 |
| **Anthropometry measures** |  |  |  |
| Weight, kg | 0.935 | (0.896,0.976) | 0.002 |
| Height, cm | 0.95 | (0.901,1.001) | 0.052 |
| BMI, kg/m^2^ | 0.837 | (0.733,0.955) | 0.008 |
| **Laboratory data** |  |  |  |
| Hemoglobin, g/L | 0.99 | (0.996,1.015) | 0.423 |
| Albumin, g/L | 0.765 | (0.638,0.917) | 0.004 |
| prealbumin, mg/L | 0.987 | (0.980,0.995) | 0.001 |
| Predialysis BUN (mmol/L) | 0.842 | (0.764,0.927) | <0.001 |
| Predialysis Creatinine(µmol/L) | 0.993 | (0.990,0.996) | <0.001 |
| Phosphorus, mmol/L | 0.396 | (0.158,0.988) | 0.047 |
| Bicarbonate, mmol/L | 1.035 | (0.967,1.108) | 0.317 |
| hs-CRP, mg/L | 1.033 | (0.976,1.093) | 0.267 |
| Kt/V | 3.467 | (0.500,24.060) | 0.208 |
| **Muscle measurement by MRI** |  |  |  |
| Thigh muscle CSA (model 1), per 1 SD | 0.58 | (0.35,0.96) | 0.035 |
| L3 trunk muscle CSA (model 1), per 1 SD | 0.37 | (0.19,0.73) | 0.004 |
| G.Med/MinM CSA(model 1), per 1 SD | 0.28 | (0.13,0.58) | 0.001 |
| G.MaxM CSA (model 1), per 1 SD | 0.28 | (0.13,0.60) | 0.001 |
| Thigh muscle PDFF (model 1), per 1 SD | 1.89 | (1.04,3.42) | 0.036 |
| L3 trunk muscle PDFF (model 1), per 1 SD | 1.71 | (1.03,2.85) | 0.04 |
| G.Med/MinM PDFF(model 1), per 1 SD | 1.44 | (0.88,2.35) | 0.143 |
| G.MaxM PDFF(model 1), per 1 SD | 1.66 | (0.98,2.80) | 0.06 |
|  |  |  |  |
| Abbreviations: CI, confidence interval; BMI, body mass index; BUN, blood urea nitrogen; hs-CRP, high-sensitive C-reactive protein; Kt/V, dialysis efficacy; L3 trunk, third lumbar trunk; G.Med/MinM, gluteus minimus and medius; CSA, muscle cross-sectional area; PDFF, proton-density fat-fraction | | | |
| We calculated the odds ratios for sarcopenia using univariate logistic regression analyses. The total number of participants was the same for all the models. | | | |

| Supplementary File S5.  Associations of muscle measurements with sarcopenia in patients undergoing hemodialysis. | | |
| --- | --- | --- |
| **Variables** | **OR (95%CI)** | ***P*** |
| **Adjusted for BMI** |  |  |
| Thigh muscle CSA, per 1 SD | 0.70 (0.41,1.20) | 0.192 |
| L3 trunk muscle CSA, per 1 SD | 0.44 (0.22,0.87) | 0.019 |
| G.Med/MinM CSA, per 1 SD | 0.31 (0.14,0.66) | 0.003 |
| G.MaxM CSA, per 1 SD | 0.32 (0.15,0.71) | 0.005 |
| Thigh muscle PDFF, per 1 SD | 1.88 (1.03,3.45) | 0.040 |
| L3 trunk muscle PDFF, per 1 SD | 1.80 (1.06,3.05) | 0.029 |
| G.Med/MinM PDFF, per 1 SD | 1.56 (0.92,2.66) | 0.098 |
| G.MaxM PDFF, per 1 SD | 1.90 (1.07,3.36) | 0.028 |
| **Adjusted for age** |  |  |
| Thigh muscle CSA, per 1 SD | 0.69 (0.40,1.20) | 0.187 |
| L3 trunk muscle CSA, per 1 SD | 0.43 (0.21,0.88） | 0.020 |
| G.Med/MinM CSA, per 1 SD | 0.31 (0.14,0.66) | 0.002 |
| G.MaxM CSA, per 1 SD | 0.34 (0.16,0.72) | 0.005 |
| Thigh muscle PDFF, per 1 SD | 1.56 (0.89,2.72） | 0.120 |
| L3 trunk muscle PDFF, per 1 SD | 1.27 (0.71,2.25) | 0.419 |
| G.Med/MinM PDFF, per 1 SD | 1.00 (0.58,1.74) | 0.999 |
| G.MaxM PDFF, per 1 SD | 1.31 (0.76,2.24) | 0.332 |
| **Adjusted for age, BMI, albumin, predialysis BUN, predialysis creatinine, and phosphorus** |  |  |
| Thigh muscle CSA, per 1 SD | 1.58 (0.69,3.62) | 0.279 |
| L3 trunk muscle CSA, per 1 SD | 1.10 (0.387,3.14) | 0.855 |
| G.Med/MinM CSA, per 1 SD | 0.66 (0.22,1.98) | 0.456 |
| G.MaxM CSA, per 1 SD | 0.81 (0.26,2.48) | 0.709 |
| Thigh muscle PDFF, per 1 SD | 1.22 (0.61,2.43) | 0.578 |
| L3 trunk muscle PDFF, per 1 SD | 1.07 (0.493,2.34) | 0.790 |
| G.Med/MinM PDFF, per 1 SD | 0.98 (0.43,2.23) | 0.956 |
| G.MaxM PDFF, per 1 SD | 1.13 (0.52,2.44) | 0.755 |
|  |  |  |
| Abbreviations: CI, confidence interval; L3 trunk, third lumbar trunk; G.Med/MinM, gluteus minimus and medius muscle; G.MaxM, gluteus maximus muscle; CSA, muscle cross-sectional area; PDFF, proton-density fat-fraction | | |
| We calculated muscle measurements odds ratios for sarcopenia using univariate and multiple logistic regression analyses. | | |
